# Supplementary material for: Organic–Inorganic Ternary Nanohybrids of Single-Walled Carbon Nanohorns for Room Temperature Chemiresistive Ethanol Detection
Source: Nanomaterials (Basel). 2020 Dec 18;10(12):2552. doi: 10.3390/nano10122552 (PMC7766401; doi:10.3390/nano10122552)
Supplement: Supplementary file 1 [file nanomaterials-10-02552-s001.pdf]

*nanomaterials*

MDPI

*Supplementary material*

# Organic–Inorganic Ternary Nanohybrids of Single-Walled Carbon Nanohorns for Room Temperature Chemiresistive Ethanol Detection

**Cornel Cobianu [1,2,*], Bogdan-Catalin Serban [1], Niculae Dumbravescu [1], Octavian Buiu [1], Viorel Avramescu [1], Cristina Pachiu [1], Bogdan Bita [1], Marius Bumbac [3,4], Cristina-Mihaela Nicolescu [4] and Cosmin Cobianu [5]**

1 National Institute for Research and Development in Microtechnologies–IMT Bucharest, 126 A Erou Iancu Nicolae str., 077190 Voluntari, Romania; bogdan.serban@imt.ro (B.-C.S.); niculae.dumbravescu@imt.ro (N.D.); octavian.buiu@imt.ro (O.B.); viorel.avramescu@imt.ro (V.A.); cristina.pachiu@imt.ro (C.P.); bogdan.bita@inflpr.ro (B.B.)
2 Science and Technology Section, Academy of Romanian Scientists, 3 Ilfov str., Sector 5, 050045 Bucharest, Romania
3 Sciences and Advanced Technologies Department, Faculty of Sciences and Arts, Valahia University of Targoviste, 13 Sinaia Alley, 130004 Targoviste, Romania; marius.bumbac@valahia.ro
4 Institute of Multidisciplinary Research for Science Technology, Valahia University of Targoviste, 13 Sinaia Alley, 130004 Targoviste, , Romania; cristina.nicolescu@valahia.ro
5 Electrical Engineering, Electronics and Information Technology Faculty, Valahia University of Targoviste, 13 Sinaia Alley, 130004 Targoviste, Romania; cosmin.cobianu@valahia.ro
* Correspondence: cornel.cobianu@imt.ro

## 1. *Fabrication of the microelectronic test structures*

The test structure, which was used for functional testing of the sensing films based on ternary nanohybrids of SWCNH's is presented in Figure S1.

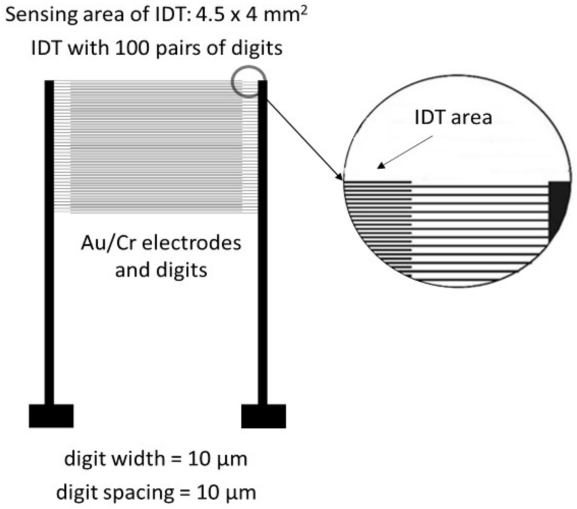


Fig. S1. Schematic layout of the silicon chip containing dual-comb interdigitated transducer. The sensing layer is deposited on the rectangle area of 4.5 x 4 $mm^2$.

It consists of a dual-comb interdigitated transducer (IDT) structure with 100 pairs of Au/Cr fingers, each with a width of 10 μm and a 10 μm spacing between two consecutive fingers. The sensing area of the IDT is equal to about 4.5 x 4 $mm^2$, and the distance between the two wider

collecting electrodes is about 0.6 mm. The advantage of such a test structure with a big number of interdigitated metal fingers is the minimization of the equivalent electrical resistance of the sensing films, which can be easier measured, even for the high resistivities of the sensing material deposited on IDT. The test structures were batch-fabricated by microelectronic technology on silicon substrates. The process consisted of thermal oxidation of a freshly cleaned silicon wafer for the growth of a $SiO_2$ layer of 1 μm thickness, which was followed by the deposition of a metal layer of Au/Cr, (100nm/10 nm). The metal patterning of IDT described above was obtained by the photolithographic process followed by etching of the metal layer from the regions where it is not needed.

## *2. Functional testing of the chemiresistive test structures based on ternary hybrids of SWCNH's*

The set-up for the automatic functional characterization of the chemiresistive test structures is presented in Figure S2.

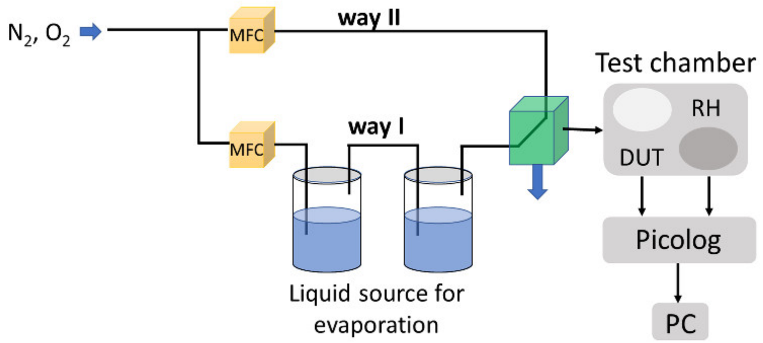


Fig. S2. Set-up for automatic testing of the ethanol chemiresistive sensors based on ternary organic-inorganic nanohybrids of SWCNH's.

It consists of the gas handling system provided with mass flow controllers (MFC) for dry oxygen and nitrogen, ethanol liquid source for controlled evaporation via air flow rate passing through the recipient, small-volume test chamber containing device under test (DUT), and an RH commercial sensor from Honeywell, PicoLog electronic interface (data logger) for collecting and analyzing the data (PICO technology), and a personal computer for recording the gas flow rate of input gases, the excitation current of the IDT and the voltage response per each second. The RH sensor is used for monitoring the relative humidity in the test chamber during the sensor exposure to the ethanol vapor for confirming that only ethanol response is measured at this stage. During sensor measurement, a Keithley 6620 constant current source is injecting current in the range (0.002-0.03) A, and the voltage drop on the IDT is measured. This voltage value is carrying the information about the electrical resistance of the chemiresistive sensor under test. All the sensing IDT test structures were measured with the sensing film kept at room temperature of 21$^{o}$C.

As shown in Figure S2, for setting and controlling an extensive range of ethanol vapor concentrations in the air of the test chamber, the synthetic dry air flow rate (obtained by combining the appropriate amounts of $N_2$ and $O_2$ flow rates) was injected via two ways, one through the ethanol liquid source (the way I) and the second directly in the chamber, (way II), under the constraint that the sum of the two flow rates to remain always constant and equal to 1 L/min in the chamber, when the airflow rate trough the way I is varied. Under these conditions, a novel method was developed for creating the desired concentration of ethanol in the air from the test chamber from

Figure S2, as it is described here. By knowing the duration of the evaporation process at a certain flow rate of air flowing through the ethanol liquid source, and measuring the mass of liquid ethanol at the beginning and the end of the process, the evaporation rate of ethanol at a temperature of 21°C as a function of airflow rate passing through liquid source was found, as shown in Figure S3. It is evident from this figure that even in the absence of airflow bubbling through liquid ethanol, there is natural evaporation of liquid ethanol determining an ethanol vapor concentration in the test chamber of 0.22 mg/min for an ambient temperature equal to 21°C. As an example, the forced evaporation due to airflow rate of 0.3 L/min through the ethanol liquid source is increased by about 200 times concerning natural evaporation, of 0.22 mg/min.

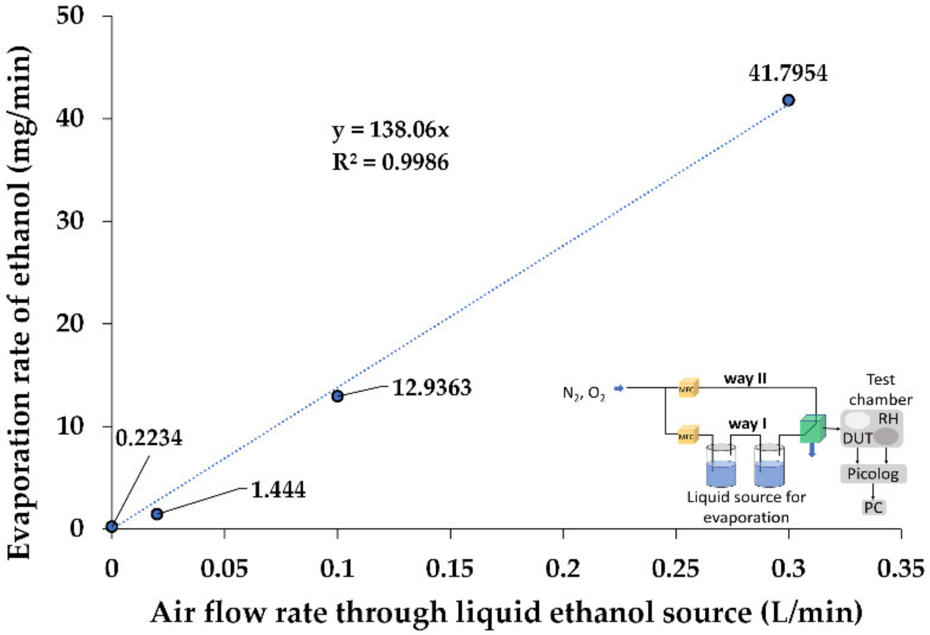


Fig. S3. Ethanol evaporation rate as a function of the airflow rate through the liquid ethanol source, at an ambient temperature of 21°C and total flow rate through ways I and II equal to 1 L/min.

With the data from Figure S3, the concentration of the ethanol vapor in the air from the test chamber for each value of the airflow rate through the way I was derived by considering the total mass of the evaporated ethanol and the total volume of the dry air passing through test chamber (cumulation of the airflow rates from the way I and way II) during the entire period of evaporation at that flow rate. Thus, a calibration plot of ethanol vapor concentration in the air from the test chamber (mg/L) as a function of the flow rate of the air passing through the liquid source (L/min) was obtained, as shown in Figure S4.

From these last two figures, one can note good linearity of the evaporation rate (Figure S3) and ethanol concentration in the test chamber (Figure S4) as a function of the air flow rate passing through the liquid ethanol source. With the support of these figures, the functional testing of the chemiresistive test structures based on ternary hybrids of SWCNH's was performed. This approach with air injection in the test chamber through the two ways was essential for the capability of the method to establish small concentrations of ethanol in air.

The experimental value of ethanol concentrations expressed in mg/L is in good agreement with the protocols used by alcohol tester for ethanol concentration in exhaled air, and therefore it has good relevance for testing such novel sensors with an eye towards future applications. If one needs the setting and control of high concentrations of ethanol in air, then, the injection of the air only through the liquid ethanol source can be used, and thus, the highest concentrations of ethanol vapor in the test chamber will be obtained for the lowest flow rates of air bubbling through the liquid ethanol.

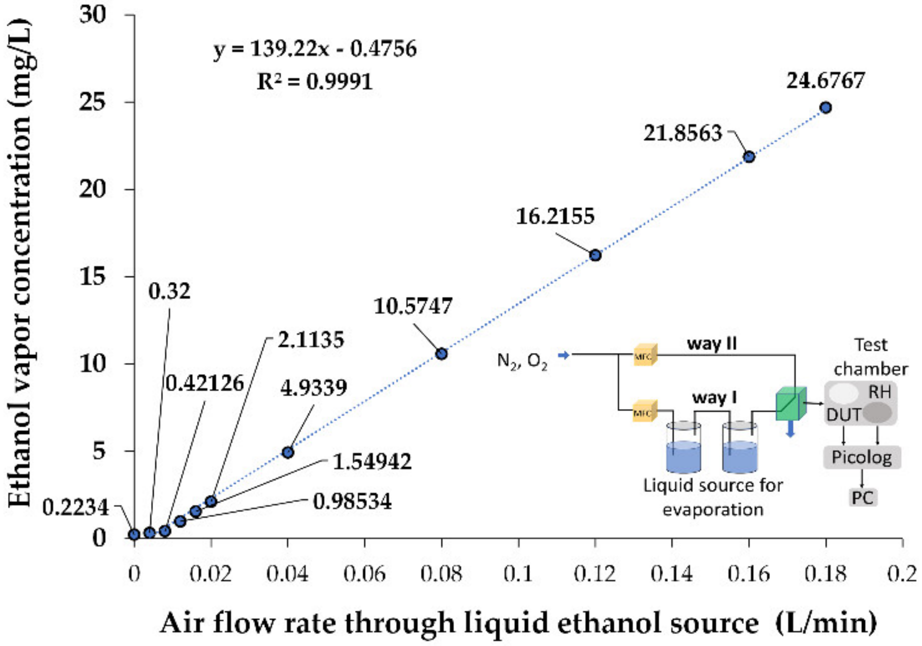


Fig. S4. The concentration of the ethanol in the air of the test chamber as a function of airflow rate through the liquid ethanol source, at an ambient temperature of 21°C and a total flow rate through ways I and II equal to 1 L/min.

### *3. Brief presentation of present status of gas sensing for VOC detection*

The commercial $SnO_2$-based ethanol chemiresistive sensors performed in thick film technology, like TGS 822, performed by Figaro company have been used most frequently in both industrial and consumer applications, but unfortunately, they are power-hungry, as they require heating of the sensing layer up to 350°C to obtain an optimum sensitivity in the range of 1-1000 ppm of alcohol in the air [1]. The use of the binary core-shell nanocomposites of $SnO_2/TiO_2$ has improved the thermal stability of the $SnO_2$ material. Still, it did not reduce the operating temperature of the chemiresistive gas sensors below 220°C [2]. Similarly, the nano-coaxial binary heterojunctions consisting of p-type $Co_3O_4$ and n-type $TiO_2$ did not reduce the ethanol detection temperature below 260°C [43].

Composites of semiconducting metal oxides and carbonic materials, like graphene-$ZnFe_2O_4$, have also reduced the operating temperature of the chemiresistive sensors for volatile organic compounds (VOC's) detection to about 275°C for a concentration of acetone equal to 10 ppm [4].

Low-temperature, chemiresistive detection of volatile organic compounds (VOC's) has been achieved by using binary nanocomposites of polyaniline and zinc oxide (ZnO), with good sensitivity, response, and recovery times when the sensing film was kept at 90°C [5], while in the case sensing films based on $TiO_2$ and poly(vinylidene fluoride), room temperature detection of ethanol concentrations higher than 150 ppm was reported [6].

The electrospun membranes made with PVP nanowires have shown ethanol sensitivity at room temperature when these were deposited as a sensing layer on the surface of a quartz crystal microbalance (QCM) and the response and recovery times were in the range of (5-500) s and (5-2700) s, respectively, depending on the concentration of the PVP in ethanol solution before spin casting [7]. The ethanol detection mechanism consists of PVP molecules acting as proton acceptors while ethanol vapors as a proton donor. However, the ethanol detection based on QCM devices has complex reading electronics and remains mainly a scientific method for investigating sensing properties of thin film-based materials.

The sensing properties of PVP as a hydrophilic polymer combined with its good adhesion to the solid substrate and its dispersing role for the carbonaceous nanomaterials in the liquid-state supported its selection in organic-inorganic hybrids for gas detection. This is the reason for selecting the PVP as a binder for our ternary composites, too.

## 4. References


1. Ivanov, P.; Llobet, E.; Vilanova, X.; Brezmes, J.; Hubalek, J.; Correig, X.; Development of high sensitivity ethanol gas sensors based on Pt-doped $SnO_2$ surfaces, *Sensors and Actuators B* **2004,** 99, 201-206.
2. Vaezi, M.R.; Shendy, K.; Ebadzadeh, T.; Synthesis of $TiO_2/SnO_2$ core-shell nanocomposites by chemical route and its gas sensing properties, *Indian J. Phys.* **2012**, *86(1)*, 9-13.
3. Liang, Y.Q.; Cui, Z.D.; Zhu, S.L. Li, Z.Y.; Yang, X.J.; Chen, Y.J.; Ma, J.M.; Design of a highly sensitive ethanol sensor using a nano-coaxial p-$Co_3O_4$/n-$TiO_2$ heterojunction synthetized at low temperature, *The Royal Society of Chemistry* **2013**, doi:10.1039/c3nr03616b.
4. Liu, F.; Chu, X.;Dong, Y.; Zhang, W.;Sun, W.; Shen, L. Acetone Gas sensors based on graphene-ZnFe2O4 composite prepared by solvothermal method. *Sensors and Actuators B* **2013**, *188*, 469-474.
5. Huang, J.; Yang, T; Wang, Y.; Wang, S.; Gas sensing performance of polyaniline/ZnO organic inorganic hybrids for detecting VOC's at low temperature, *Journal of Natural Gas Chemistry* **2011**, *20* , 515-519.
6. Marook , M.; Hawkins, P.; A rapidly-responding sensor for benzene, methanol and ethanol vapours based on films of titanium dioxide dispersed in a polymer operating at room temperature. *Sensors and Actuators* B **2001**, *75*, 197-202.
7. Aria, M. M.; Irajizad, A.; Astaraei, F.R.; Shariatpanahi, S.P.; Sarvari, R.; Ethanol sensing properties of PVP electrospun membranes studied by quartz crystal microbalance, *Measurement* **2016**, *78*, 283-288.


© 2020 by the authors. Licensee MDPI, Basel, Switzerland. This article is an open access article distributed under the terms and conditions of the Creative Commons Attribution (CC BY) license (http://creativecommons.org/licenses/by/4.0/).
